# Primary gastric tuberculosis was misdiagnosed as submucosal mass: A rare case report and review of literature

**DOI:** 10.1097/MD.0000000000034433

**Published:** 2023-07-28

**Authors:** Shuyan Feng, Yi He, Chi Zhang, Dandan Chen, Jihong Zhong, Shuo Zhang

**Affiliations:** a Zhejiang Chinese Medical University, Hangzhou, China; b Sir Run Run Shaw Hospital, Zhejiang University, Hangzhou, China; c The Second Affiliated Hospital of Zhejiang Chinese Medical University, Hangzhou, China.

**Keywords:** endoscopic ultrasound, endoscopy, gastric tuberculosis, pathological section, submucosal mass

## Abstract

Tuberculosis of stomach is the rarest form of the tuberculosis family. It is not only lacks specificity in clinical symptoms, but also lacks specificity in auxiliary examination results such as upper gastrointestinal barium meal and gastroscopy. In addition, gastric tuberculosis can coexist with gastric ulcer or gastric cancer, which is difficult to confirm the diagnosis and easy to misdiagnose.

**Case summary:**

We report a patient who presented with gastric discomfort as the main symptom and was diagnosed with gastric submucosal mass at a local hospital several months ago. For further endoscopic treatment, the patient came to our hospital for hospitalization.

**Conclusion:**

We performed endoscopic ultrasonography and histopathological biopsy for the patient, and found that the “submucosal mass” was actually a gastric tuberculosis lesion. After confirming the diagnosis of gastric tuberculosis, we transferred the patient to a tuberculosis specialist hospital for antituberculosis treatment.

Through a series of literature review, we rediscuss the diagnosis and differential diagnosis of gastric tuberculosis, aiming at improving the understanding of gastroenterologists to this disease, so as to timely diagnose and treat patients with gastric tuberculosis.

## 1. Introduction

Gastrointestinal tuberculosis is a relatively rare type of tuberculosis found in the human body, and it is generally secondary to pulmonary tuberculosis, bone tuberculosis, urinary system tuberculosis, and pelvic tuberculosis. Gastrointestinal tuberculosis can occur in various parts of the gastrointestinal tract, among which ileocecal tuberculosis is the most common, and gastric tuberculosis is the rarest, with few clinical reports.^[1]^ Patients with gastric tuberculosis not only lack specific clinical symptoms but also lack specificity in the results of auxiliary examinations, such as upper gastrointestinal barium meal and gastroscopy. In addition, gastric tuberculosis can coexist with other stomach diseases, such as gastric ulcers or gastric cancer, making the diagnosis of gastric tuberculosis more difficult. Therefore, clinical cases of gastric tuberculosis misdiagnosed as other gastrointestinal diseases are quite common.^[2]^ The diagnosis of most patients with gastric tuberculosis often depends on gastroscopic tuberculosis tissue biopsy and histopathology. Clinicians can confirm the diagnosis of gastric tuberculosis by finding caseous epithelioid granuloma or acidophilic bacilli in pathological sections. The tuberculin test and positive antituberculosis antibody also have certain auxiliary diagnostic value.^[3]^ We encountered a middle-aged woman who had been diagnosed at a local hospital with a submucosal mass on the lesser curvature of the stomach. The possibility of submucosal masses, such as gastric stromal tumors (gist) or gastric lipomas, was ruled out by endoscopic ultrasonography, and granulomatous tissue and a small amount of acidophilus were found in the lesion through tissue biopsy. Combined with the positive tuberculin test of the patient, the rare disease of gastric tuberculosis was confirmed.

## 2. Case presentation

### 2.1. Chief complaints

The chief complaint of the patient was 1 year of epigastric pain.

### 2.2. History of present illness

A 50-year-old middle-aged woman had a history of rheumatoid arthritis and had undergone cesarean sections in 1995 and 2002. She had no history of hypertension, diabetes, or other internal diseases, and she did not have a history of drug or food allergies. In January 2022, the patient developed epigastric pain for the first time without obvious cause. The symptoms of abdominal pain were aggravated after eating, and they were sometimes mild and sometimes severe. The patient went to the Department of Gastroenterology of local hospital A. At that time, the patient underwent upper gastrointestinal endoscopy and was found to have a shallow ulcer in the duodenal bulb, which was in scar stage S2. In addition to duodenal ulcers, the patient also suffered from chronic superficial gastritis and erosion-like changes on the surface of the gastric antrum. At that time, the patient also had an abdominal ultrasound examination, and the report showed that there was a small amount of cholesterol crystals in the gallbladder wall. Doctors believed that chronic gastritis and duodenal ulcer were the main causes of abdominal pain. After taking proton pump inhibitors (PPI) for a period of time as directed by the doctor and improving her bad eating habits, the symptoms of epigastric pain improved slightly, but in the following months, the symptoms of epigastric pain repeatedly occurred. In June 2022, the patient’s upper abdominal pain worsened, and she went to a local hospital and received another upper gastrointestinal endoscopy. The gastroscopy reexamination indicated that the patient still had inflammation and erosion in the gastric antrum, but that the duodenal ulcer discovered 5 months prior had healed after PPI treatment. In addition, a new small polyp was found and removed by the endoscopist. The pathological report of the biopsy tissue taken from the gastric antrum erosion indicated mild chronic superficial gastritis, and the small polyp was benign hyperplasia [HP (−)]. In addition to an upper gastrointestinal endoscopy, the patient underwent enhanced computed tomography (CT) examination of the whole abdomen in June 2022. Several enlarged lymph nodes were found in the hepatogastric space, and there was a local distinct lesion of the lesser curvature of the gastric body with a diameter of approximately 1 cm, which was not mentioned in the gastroscopy report. Compared to the report from January 2022, the abdominal ultrasonography in June 2022 showed that the lymph nodes between the left lobe of the liver and the stomach were enlarged, potentially excluding the possibility of bacterial infection, inflammation, and even malignant tumors.

**Figure 1.**
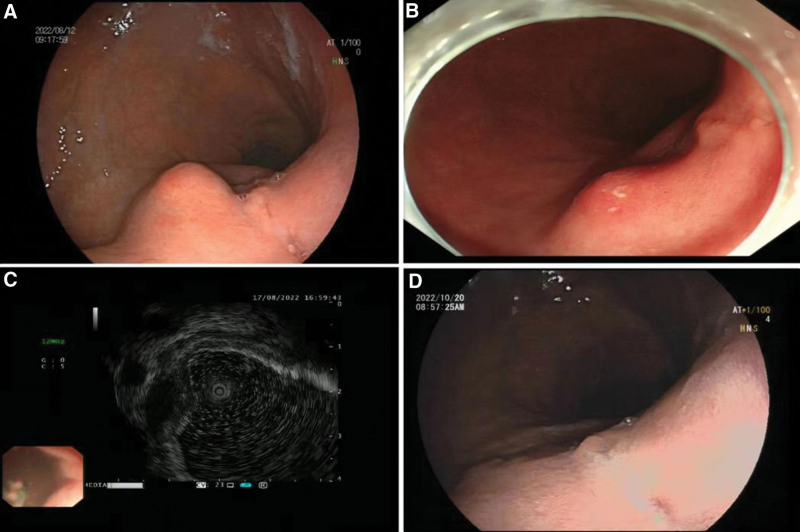
Endoscopic findings: (A) gastroscopy showing smooth mucosal protrusion on the posterior wall of the lesser curvature of the gastric body. (B) Gastroscopy showing mucosal protrusion on the posterior wall of the lesser curvature of the gastric body, redness on the surface, a small amount of white mucus attached, and scar changes of a longitudinal ulcer beside the lesion. (C) EUS showing mixed echogenic lesions, mainly hypoechoic. (D) Gastroscopy after treatment showing slit-like changes on the posterior wall of the lesser curvature of the gastric body, and the lesion was significantly improved compared to prior gastroscopy observations.

The symptoms of epigastric discomfort worsened in early August 2022. For better diagnosis and treatment, the patient decided to go to hospital B, the largest hospital in the city she lived in. The patient underwent relevant auxiliary examinations at hospital B, and the abdominal enhancement CT report was similar to that reported in June 2022, suggesting that the local wall of the lesser curvature of the gastric body was thickened, with a diameter of approximately 1 cm, and surrounded by multiple enlarged lymph nodes. An abdominal B-ultrasound showed multiple lymph node enlargements in front of the cardia. On August 12, 2022, the patient underwent gastroscopy at hospital B, which identified a smooth protruding lesion (Fig. 1A) with a diameter of approximately 1.0 cm in the posterior wall of the lesser curvature of the stomach. The lesion, which was slightly soft to the touch of the biopsy forceps, was considered a submucosal lesion, and the patient was advised to undergo further endoscopic ultrasonography.

**Figure 2.**
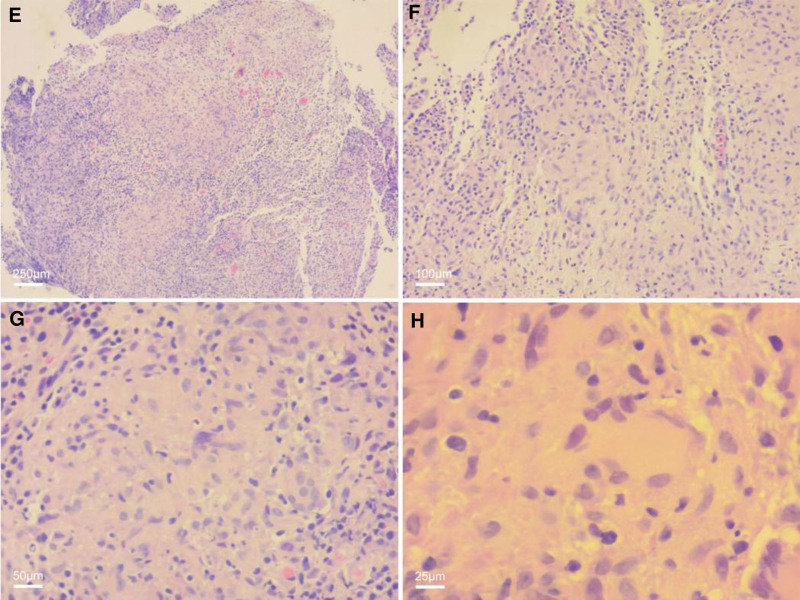
Gastric tuberculosis lesions at different magnifications, including (A) 40×, (B) 100×, (C) 200×, and (D) 400×. Histopathological examination (HE staining) showing chronic moderately active mucosa with necrotizing granulomatous inflammation, numerous multinucleated giant cells, and occasional eosinophilic bacilli.

On August 16, 2022, the patient came to our hospital for endoscopic treatment due to a submucosal mass. We performed an abdominal physical examination on the patient with the following assessment: flat abdomen; no gastrointestinal type or peristaltic wave was observed; no varicose veins were observed in the abdominal wall; soft abdomen; mild tenderness in the middle and upper abdomen; no rebound tenderness; Murphy sign and shifting dullness were negative; and bowel sounds occurred approximately 4 times/min. After excluding the relevant surgical contraindications, the patient underwent painless gastroscopy at our hospital on August 17, 2022. We also observed a lesion approximately 1.2 cm in diameter in the posterior wall of the upper part of the gastric body of the patient. Unexpectedly, this protuberant lesion was greatly changed from the shape reported by the gastroscope performed at hospital B 5 days prior (Fig. 1B), which indicated a smooth lesion with a clean surface, no fluid attachment, and no surrounding scar tissue. However, observations at our hospital 5 days later indicated that the protuberant lesion surface was not smooth and was red in color, and a small amount of white mucus was observed. When the lesion was touched with biopsy forceps, the texture was soft and immobile, and it was completely different from hard and movable submucosal protrusions, such as gastric stromal tumors. When the lesion was flushed with water, a small amount of white mucus was observed flowing out of it, prompting a preliminarily diagnosis of an abscess lesion. In addition, we also observed a longitudinal scar lesion adjacent to this distinct lesion. To determine the nature of the lesion, endoscopic ultrasonography was performed on the patient. In the endoscopic field of view, the echo of the distinct lesion was mixed, mainly hypoechoic, measuring approximately 1.45 × 0.75 cm with clear borders (Fig. 1C). Three biopsies of this lesion and 1 biopsy of the adjacent scar tissue were collected. Considering that multiple enlarged lymph nodes were previously reported on the patient’s abdominal CT and abdominal ultrasound, we began to suspect the possibility of gastric tuberculosis. Therefore, the patient was subjected to the tuberculin test and antituberculosis antibody tests. The results verified our conjecture that the tuberculin test was positive and tuberculosis antibody was negative, indicating that tuberculosis infection could not be ruled out. One week later, the pathological report of the lesion tissue indicated that the mucosa was chronic and moderately active with necrotizing granulomatous inflammation, a large number of multinucleated giant cells, and occasional eosinophilic bacilli (Fig. 2). Combined with all the above results, we confirmed the diagnosis of gastric tuberculosis and recommended that the patient be admitted to the tuberculosis hospital for special treatment. Based on the patient’s follow-up, the patient is currently receiving quadruple antituberculosis treatment (rifampicin, isoniazid, ethambutol, and pyrazinamide) in the infectious disease hospital for 1 year. After 2 months of antituberculosis treatment, the patient returned to local hospital B for gastroscopy, and the lesion in the stomach was significantly improved (Fig. 1D). We advised the patient to undergo upper gastrointestinal examination in 6 months to review the changes in the gastric lesion.

In March 2023, the patient’s gastroscopy showed complete healing of the lesion.

### 2.3. History of past illness

The patient had a history of rheumatoid arthritis and had undergone cesarean sections in 1995 and 2002. She had no history of hypertension, diabetes or other internal diseases, and the patient did not have a history of drug or food allergies.

### 2.4. Physical examination

The physical examination made the following assessments: flat abdomen; no gastrointestinal type or peristaltic wave was observed; no varicose veins were observed in the abdominal wall; soft abdomen; mild tenderness in the middle and upper abdomen; no rebound tenderness; Murphy sign and shifting dullness were negative; and bowel sounds occurred approximately 4 times/min.

### 2.5. Laboratory examinations

The tuberculin test was positive, and the tuberculosis antibody was negative. The pathological reports showed that the mucosa was chronic and moderately active with necrotizing granulomatous inflammation, a large number of multinucleated giant cells, and occasional eosinophilic bacilli (Fig. 2).

### 2.6. Imaging examinations

Abdominal CT and abdominal ultrasound revealed multiple enlarged lymph nodes.

## 3. Multidisciplinary expert consultation

Because this patient did not have other systemic diseases, multidisciplinary experts were not invited for discussion.

## 4. Final diagnosis

The patient was diagnosed with gastric tuberculosis.

## 5. Treatment

The patient is currently receiving quadruple antituberculosis treatment (rifampicin, isoniazid, ethambutol, and pyrazinamide) in the infectious disease hospital for 1 year.

## 6. Outcome and follow-up

After 2 months of antituberculosis treatment, the patient returned to local hospital B for gastroscopy, and the lesion in the stomach was significantly improved (Fig. 1D). In March 2023, the patient’s gastroscopy showed complete healing of the lesion.

## 7. Discussion

Intestinal tuberculosis and tuberculous peritonitis are the most common types of tuberculosis in the digestive tract followed by liver tuberculosis and gastric tuberculosis, which is rare with only a few clinical reports.^[4]^ Gastric tuberculosis mostly occurs in the pylorus and lesser curvature of the prepyloric region, and a few lesions may occur in the gastric body or greater curvature.^[5]^ The etiology of gastric tuberculosis is not clear, but it is currently believed that it may be related to destruction of the mucosal barrier, decreased gastric juice secretion, weakening of the bactericidal effect of gastric juice after gastric mucosal injury, insufficient gastric motility, decreased gastric emptying rate, and increased *Mycobacterium tuberculosis* length of stay in the stomach to allow sufficient time to form a tuberculosis focus.^[6]^ At present, some studies have suggested that the reduction in intragastric immunity caused by the absence of lymphoid follicles in the gastric wall may also be 1 of the causes of gastric tuberculosis.^[7]^ Most gastric tuberculosis lesions are secondary to primary tuberculosis of the lung, intestine, bone, and other parts of the body.^[8]^ The secondary infection mechanisms of gastric tuberculosis are as follows: *M tuberculosis* reaches the stomach through blood circulation or lymph circulation to form the focus of tuberculosis; tuberculosis lesions of adjacent organs spread and directly penetrate or invade the stomach to form gastric tuberculosis lesions; and when gastric ulcers or gastric cancer lesions exist, the gastric mucosa is damaged, gastric acid is reduced, and the bactericidal power of gastric juice is decreased, allowing *M tuberculosis* to invade all layers of the stomach and form tuberculosis lesions.^[9]^ The diagnosis of gastric tuberculosis is difficult and easy to misdiagnose because most patients with gastric tuberculosis often have no specific clinical manifestations. In addition to the general symptoms of afternoon low-grade fever, night sweats, emaciation, fatigue, anemia, and other symptoms of active tuberculosis, only some patients have manifestations similar to chronic gastritis and gastric cancer. Epigastric pain is the most common symptom in patients with gastric tuberculosis, with varying degrees of pain; in addition, abdominal tenderness is often located to the right below the xiphoid process, and sometimes peptic ulcers, such as hematemesis, and black stool may also occur.^[10]^ When gastric tuberculosis is complicated with pyloric obstruction, nausea and vomiting are the main symptoms, in which vomiting is aggravated in the afternoon and evening. Moreover, the vomit does not contain bile, and abdominal distension is alleviated after vomiting. Physical examination can include epigastric stomach type, peristaltic wave, and tremor sound. Some patients are often misdiagnosed with gastric cancer due to a palpable epigastric mass and positive fecal occult blood.^[11]^ There are no typical features of upper gastrointestinal barium meal and endoscopic local manifestations. In addition, gastric tuberculosis often exists with gastric ulcers and gastric cancer, making the diagnosis more complicated and easier to misdiagnose. According to the morphological characteristics of endoscopic lesions, gastric tuberculosis can be divided into ulcerative type, protuberant type, miliary nodular type, and inflammatory infiltrating type. More than 80% of gastric tuberculosis is ulcerative, and some patients with gastric tuberculosis can even show giant gastric ulcers.^[12]^ The gold standard for the diagnosis of gastric tuberculosis is to find the presence of a caseous epithelioid granuloma or acidophilus under the pathological examination of the biopsy tissue. For the treatment of gastric tuberculosis, antituberculosis therapy is generally used. For patients with a definite diagnosis of gastric tuberculosis, systematic antituberculosis treatment should be performed as early as possible.^[13]^ Guidelines recommend the use of 2 to 4 first-line antituberculosis drugs; the preferred drugs are streptomycin and isoniazid, with a course of 12 to 18 months. For patients with acute massive hemorrhage who fail to respond to conservative treatment, those with perforation obstruction, those with abdominal masses that are difficult to distinguish from malignant tumors, and those with coexisting gastric tuberculosis, they should be treated as early as possible.^[14]^

In the present study, we provided a detailed analysis of a gastric tuberculosis case, involving a 50-year-old middle-aged woman with a history of immune arthritis and autoimmune dysfunction. The present case had a history of antral erosion caused by chronic gastritis for many years and complicated with a duodenal ulcer, indicating an increased likelihood of tuberculosis bacteria invading from the digestive tract. Therefore, this patient belonged to the high-risk group for gastric tuberculosis. The main clinical symptom of the patient was upper abdominal pain. Because the patient was complicated with chronic gastritis, antrum erosion, and duodenal ulcer as well as had no specific tuberculosis symptoms, such as fatigue, low-grade fever, night sweats, and no previous history of tuberculosis, we did not consider the possibility of tuberculosis at the beginning but attributed the cause of upper abdominal pain to gastritis and ulcers. In the physical examination, the patient had no other positive pathological signs, except for mild tenderness in the upper abdomen. The initial gastroscopy report of the patient at local hospital A only showed gastritis and duodenal ulcers in the patient’s stomach, and the ulcer was healed in the second gastroscopy report after PPI treatment. However, the patient’s upper abdominal pain did not significantly improve, which first allowed us to consider that the patient’s upper abdominal pain was caused by other organs. However, at that time, the patient received abdominal enhanced CT and ultrasound examination, which showed no significant abnormality, and lesions of other organs were excluded. In the gastroscopy report of hospital A in June 2022, the endoscopist found a small polyp and removed it, but these small polyps usually do not cause symptoms of upper abdominal pain. The abdominal CT examination in June 2022 found many newly enlarged lymph nodes in the abdomen as well as a bulge in the lesser curvature of the stomach, but these changes were not evident endoscopically. In August 2022, the endoscopic review results of hospital B reported this distinction. At that time, the surface of this distinct lesion was smooth and looked like a submucosal distinct mass. However, the rapid progression of the disease made us suspicious of this diagnosis. The mass was soft in texture and hard to push with biopsy forceps, unlike a submucosal mass. Finally, the patient came to our hospital and underwent endoscopic ultrasound examination. The lesion showed mixed hypoechogenicity with a size of 1.45 × 0.75 cm under endoscopic ultrasonography, which was considered to be an abscess, and this lesion had changed greatly from the report of hospital B 1 week prior, with an unsmooth surface, white mucus coverage, and white fluid outflow with water impact. In addition, there was a scar-like change adjacent to the protuberant lesion. Considering the enlarged celiac lymph nodes and the abscess lesion of the lesser curvature of the stomach, we considered the possibility of gastric tuberculosis. The positive tuberculin test and the final pathological report of the biopsy tissue confirmed our conjecture. The submucosal mass was a rather rare gastric wall abscess caused by gastric tuberculosis, which is important to distinguish from other diseases characterized by submucosal masses, such as early gastric cancer, leiomyoma, and stromal tumor. Endoscopic ultrasound is of great value in the diagnosis of gastric tuberculosis lesions and can aid in the understanding of the internal echo structure of the mass and the relationship between the various layers of the gastric wall. It is worth exploring the patient’s negative antituberculosis antibody test; this result may have been due to the patient’s rheumatoid arthritis and autoimmune dysfunction, which do not produce the corresponding antituberculosis antibody. antituberculosis antibodies also have a certain probability of false negatives. After 2 months of antituberculosis treatment, the patient was reexamined by upper gastrointestinal tract gastroscopy at local hospital B, which showed that the intragastric lesion had significantly improved, which indirectly suggested that oral antituberculosis drug therapy is effective in the treatment of gastric tuberculosis.

## 8. Conclusion

We report a rare case of protuberant type complicated with ulcerative gastric tuberculosis. After multiple examinations of the upper digestive tract, we found that in a short period of half a year, the patient successively developed protuberant lesions on the lesser curvature, ulcer scar lesions, enlarged abdominal lymph nodes, and other symptoms. Through endoscopic ultrasound examination, pathological report of tissue biopsy, and clinical symptoms of the patient, we confirmed the diagnosis of gastric tuberculosis. In the present case report, we also reviewed the main points of diagnosis and differential diagnosis of gastric tuberculosis through a literature study, aiming to help clinicians expand their understanding of gastric tuberculosis to diagnose and treat patients with gastric tuberculosis in a timely manner.

## Author contributions

**Conceptualization:** Jihong Zhong, Shuo Zhang.

**Writing – original draft:** Shuyan Feng, Yi He, Chi Zhang, Dandan Chen.

**Writing – review & editing:** Shuyan Feng, Chi Zhang, Shuo Zhang.
